# Pitfalls in the design and analysis of paediatric clinical trials: a case of a ‘failed’ multi-centre study, and potential solutions

**DOI:** 10.1111/j.1651-2227.2008.01048.x

**Published:** 2009-02

**Authors:** Johanna H van der Lee, Michael WT Tanck, Judit Wesseling, Martin Offringa

**Affiliations:** 1 Department of Paediatric Clinical Epidemiology, Emma Children's Hospital, Academic Medical Centre, University of AmsterdamThe Netherlands; 2 Department of Clinical Epidemiology, Biostatistics and Bioinformatics, Academic Medical Centre, University of AmsterdamThe Netherlands; 3 Department of Paediatrics, Red Cross HospitalBeverwijk, The Netherlands; 4 Department of Neonatology, Emma Children's Hospital, Academic Medical Centre, University of AmsterdamThe Netherlands

**Keywords:** Clinical trials, Paediatrics, Sample size, Statistics

## Abstract

Aim: To increase awareness of possible pitfalls in the design and analysis of a multi-centre randomized clinical trial and to give an overview of alternative study designs and their consequences for power analyses in case of limited availability of trial participants.

Methods: Investigation of the assumptions in the power calculation and re-analysis of the original data of a ‘failed’ trial on the effect of dexamethasone on the duration of mechanical ventilation in young children with respiratory syncytial virus infection. Use of ‘boundaries approach’ is explored using the data from this trial. A comprehensive overview of the various modern solutions for the design of a subsequent trial in this field is given.

Results: Two frequent major deficiencies of trial design and data analysis are reviewed in depth, i.e. too optimistic assumptions for the sample size calculation and failure to adjust for centre effects.

Conclusion: Critical review of trial assumptions and if necessary sample size recalculation based on an internal pilot by a data monitoring committee is recommended to maximize the probability of obtaining conclusive results.

## INTRODUCTION

Just as analytic reports of notable patient cases can provide insight into the pathophysiology of a disease or lead to the recognition of side-effects of a drug (1), the report of a single clinical trial can provide insight into methodological issues that may not be obvious at first sight. In this paper we use the information from a single randomized clinical trial (RCT) to discuss methodological issues that may not be obvious at first sight, but may have serious implications for the usefulness of the results of the trial in clinical practice, and may guide future study design in this field.

The case came to our attention through our methodology-consultation service in the Emma Children's Hospital (ECH). We think that this trial on the management of ventilated infants with respiratory syncytial virus (RSV) infection is a case in point for many other trials with comparable challenges, both in paediatrics and in other medical specialties. We will only pay attention to medical details as far as is necessary to understand the methodological issues.

*Case:* From December 1997 to March 2001, data were collected for a multi-centre randomized placebo-controlled trial in the Netherlands to evaluate the efficacy of intravenous dexamethasone in young patients mechanically ventilated for respiratory syncytial virus lower respiratory tract infection (RSV-LRTI) (2). Randomization was stratified by centre. The number of patients to be included was calculated based on the notion that a between groups reduction in duration of mechanical ventilation of 1.5 days was clinically relevant and on an estimated Standard Deviation (SD) of the mean duration of ventilation of 2 days. This calculation, setting the type I error rate (α) at 5% and the type II error rate (β) at 20% (i.e. power 80%), resulted in a required sample size of two groups of 30 patients. However, although the number of included patients who eventually completed the trial (n = 82) was sufficient, and the mean difference between the placebo group and the dexamethasone group was deemed clinically relevant (1.6 days), the 95% Confidence Interval (95% CI) was not narrow enough to reach statistical significance (−0.8 to 3.8 days) (2). This 95% CI indicates that dexamethasone may reduce the duration of mechanical ventilation with almost 4 days or it may extend its duration with almost 1 day. It means that, although the difference is not statistically significant, the possibility of a clinically relevant beneficial effect of dexamethasone has not been excluded. This result could be described as ‘no evidence of effect’ (3). And so, in spite of an apparently adequate power calculation, the trial did not yield results on which clinicians could base their decision to treat RSV-LRTI patients with dexamethasone or not. The authors of the report concluded that ‘the results of this trial show that dexamethasone does not lead to a shorter duration of mechanical ventilation in patients with RSV-LRTI’ (2). Thus their – incorrect – interpretation was ‘evidence of no effect’.

During the data collection phase of this trial, the results of another study were published, in which the existence of two clinically different subgroups of patients with RSV-LRTI was postulated, based on the extent of gas-exchange anomalies during the first hours of admission in the Pediatric Intensive Care Unit (PICU) (4). Patients were classified as having RSV bronchiolitis if PaO_2_/FiO_2_ > 200 mmHg and/or mean airway pressure ≤ 10 cmH_2_O and as having RSV pneumonia if PaO_2_/FiO_2_≤ 200 mmHg and mean airway pressure > 10 cmH_2_O (5). This led to a post-hoc analysis by Van Woensel et al. to see whether dexamethasone has a differential effect in these subgroups (2). The data on arterial blood–gas analysis could be retrieved in 80 patients. In the bronchiolitis subgroup (n = 39) a statistically significant and clinically relevant reduction in days on ventilator was found in favour of dexamethasone (mean difference 4.3 days; 95% CI 0.8–7.8 days), whereas in the pneumonia subgroup (n = 41) the mean difference, although not statistically significant, was in favour of placebo (difference −0.7 days; 95% CI −3.6 to 2.2 days). The authors found these results difficult to interpret, and they proposed to do further prospective studies on this topic.

**Figure 1:**
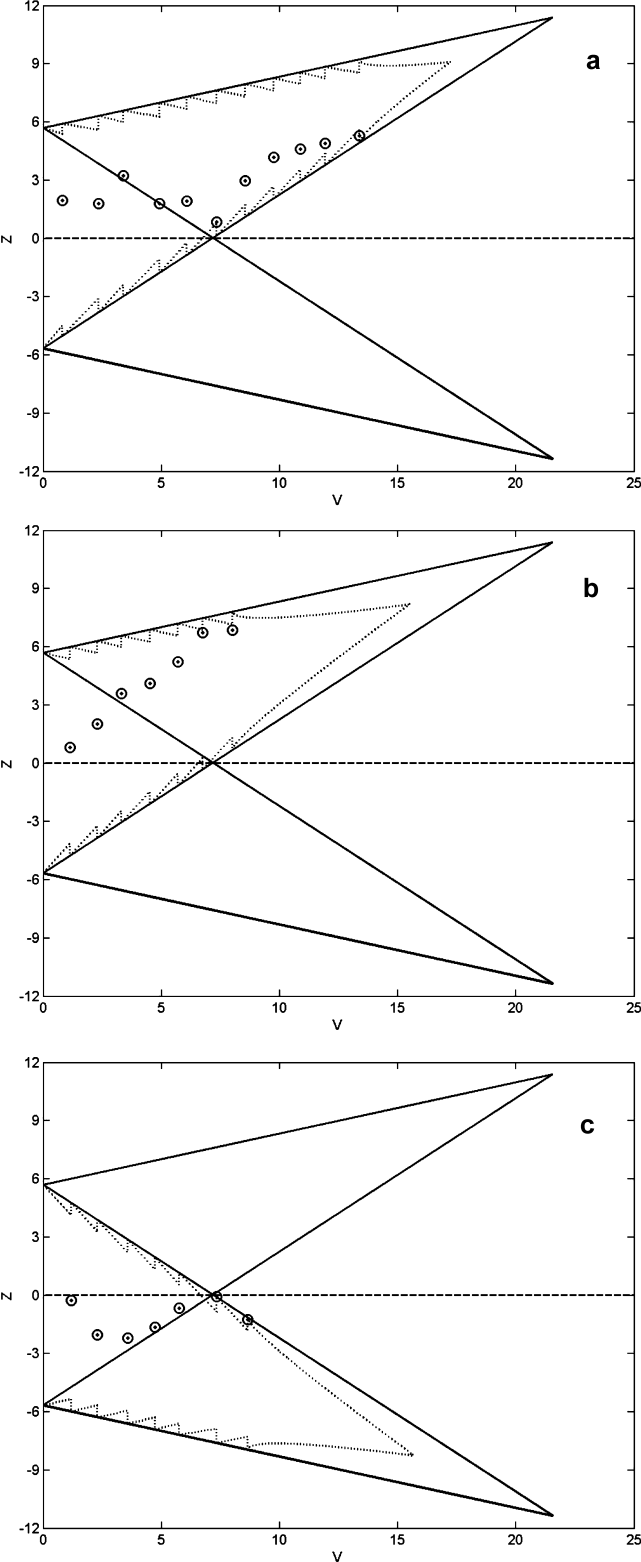
Double triangular test designed to have 80% power to detect a significant (two-sided α of 0.05) difference of 1.5 days between the two treatments assuming a standard deviation of 2 days with the sample path based on inspection intervals of five patients (a) after 11 inspections (n = 55) in the total patient population; (b) after 7 inspections (n = 35) in the bronchiolitis subgroup; (c) after 7 inspections (n = 35) in the pneumonia subgroup. Figures (b) and (c) represent post-hoc analyses without appropriate correction for multiple testing.

**Figure 2:**
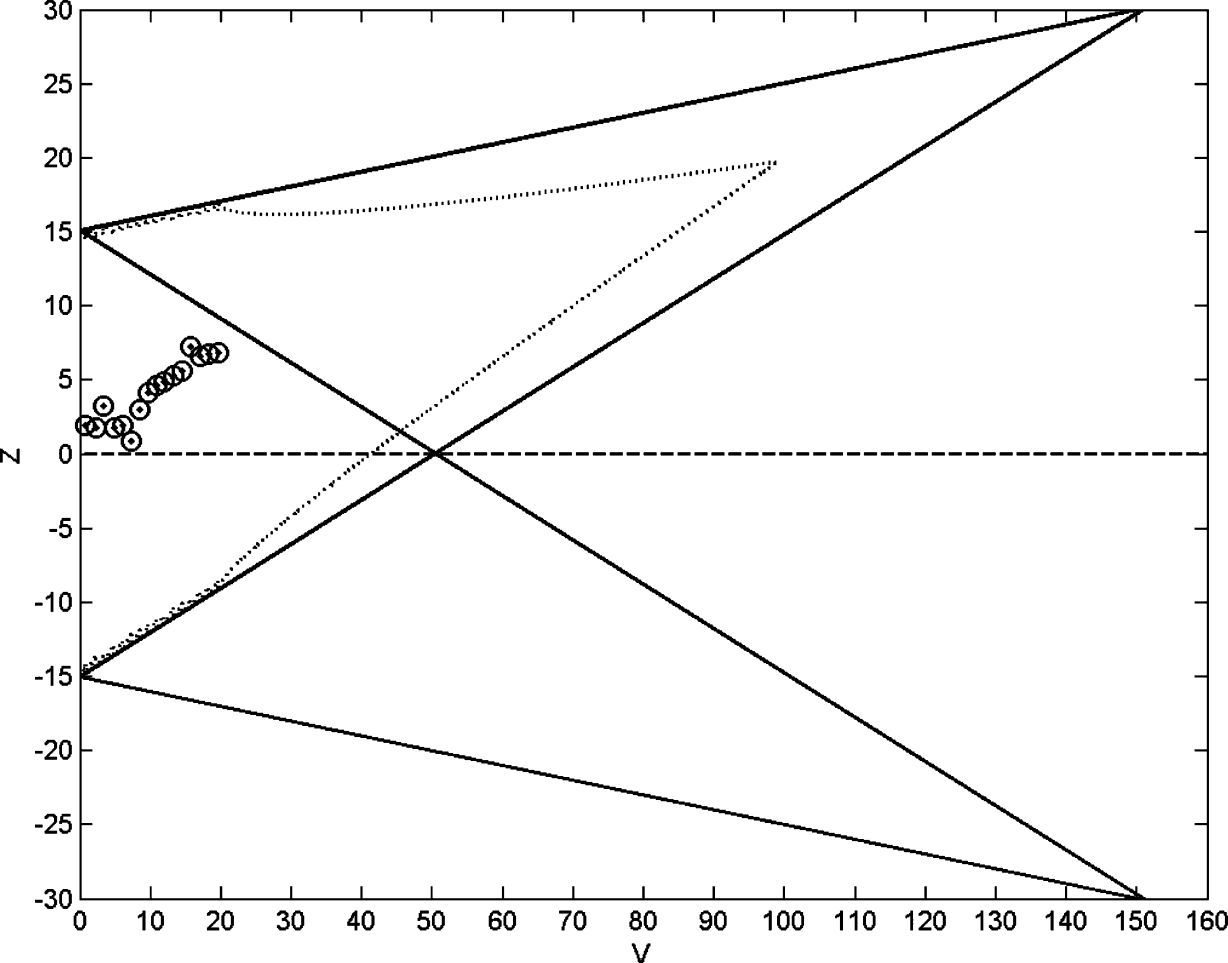
Double triangular test designed to have 80% power to detect a significant (two-sided α of 0.05) difference of 1.5 days between the two treatments assuming a standard deviation of 5.3 days with the sample path based on inspection intervals of five patients after 16 inspections (n = 80) in the total patient population.

In the preparation phase of this subsequent research, our Department of Paediatric Clinical Epidemiology was consulted. One of the questions was whether a triangular test approach would be more efficient than a fixed sample approach. In a triangular test the sample size is not fixed at the start of the trial, but it is derived empirically during the trial as the amount of information accumulates. The boundaries for stopping inclusion of patients in the trial are set in advance, based on assumptions for α, β and the expected effect size Θ, i.e. the minimal clinically relevant difference divided by its SD (6). These boundaries form a triangle in a graph defined by V on the *X*-axis and Z on the *Y*-axis. V is a measure of the amount of information gathered so far and Z is a measure of the effect size. The boundaries are defined by the formulae Z = a + cV and Z =−a + 3cV. In case of a double triangular test, in which both possibilities of better and worse outcome of the experimental compared to the control treatment are investigated, two pairs of boundaries are plotted symmetrically around the horizontal axis. The boundaries are then also defined by Z =− a − cV and Z = a − 3cV. This is illustrated in Figures 1 and 2. The values of Z and V are plotted in this graph after the outcome of each individual or of a group of patients. The resulting series of dots is called the sample path. Each analysis leads to a decision either to continue the trial as long as the sample path is between the boundaries or to stop including patients when one of the boundaries is crossed. Crossing of the upper boundary of the single triangular test leads to the conclusion that the experimental treatment is superior; crossing of the first part of the lower boundary leads to the conclusion that the experimental treatment is inferior, crossing of the (dashed) right part of the lower boundary leads to non-rejection of the null-hypothesis. Crossing of the upper or lower boundary of the double triangular test leads to the conclusion that the experimental treatment is superior or inferior, respectively; crossing of the boundaries between both triangles leads to non-rejection of the null-hypothesis. Each analysis also leads to adaptation of the boundaries, which leads to the so-called Christmas tree shape of the boundaries. The apex of the triangles designates the maximum value of V, and thus can be converted into the maximum number of patients that have to be included in the trial if none of the boundaries has been crossed before this amount of information is assembled (6,7). The computer program PEST can be used to plot the boundaries and sample path and to calculate the point estimate and its 95% CI (8). If the assumption for the expected effect size was correct, the power and type I error rate are maintained throughout this procedure. In a single triangular test the power to detect superiority of the experimental treatment is set at a specified percentage; the power to detect inferiority of the experimental treatment is much lower.

Considering the challenges posed by this case, we asked ourselves the following questions:

1. What was the cause for the non-conclusive results in the first trial?
2. What can be done to prevent non-conclusive results like this?
3. Could the same results have been obtained more efficiently, i.e. including fewer patients, with a ‘boundaries approach’?
4. What is the most efficient way to answer the research question concerning the effectiveness of dexamethasone in reducing the duration of mechanical ventilation in the two subgroups of children with RSV bronchiolitis and RSV pneumonia?

## METHODS

To answer the first question, the assumptions used in the power calculation were compared with the empirically derived data from the trial. If the assumptions were right, there was still a chance of 20% of a type II error.

The second question is answered by recalculation of the variance in the former trial and by referring to the literature about sample size calculations and the conduct of randomized clinical trials.

Subsequently, to answer question no. 3, double triangular tests were performed for the entire study population and for both subgroups using the raw data from the trial. This was done first with the assumptions of the trial, then with the empirical measures of variance.

To answer the fourth question alternative approaches to study design are considered and their consequences for the necessary number of patients to be included are estimated.

## RESULTS

### 1 Cause of the non-conclusive results

The only truly uncertain factor of the assumptions in the power calculation was the SD of the duration of mechanical ventilation in days. This was originally assumed to be 2 days, based on the findings in a subgroup in an earlier, single-centre trial (9). However, when analysing the data, this was found to be an underestimate, since the empirical SD in the study population in this multi-centre trial turned out to be 5.3 days. Application of this value for the SD in the power calculation would have resulted in a sample size of 196 instead of 30 per intervention group.

Estimations of sample sizes for various design options and assumptions are shown in Table 1. It might be that the large variance in the main outcome of the entire study population was due to differences in the disease subgroups. However, the empirical SDs were 4.6 and 5.9 days in the pneumonia and bronchiolitis subgroups, respectively.

**Table 1:** Estimated total sample sizes using a fixed sample size design and the triangular test approach in a subsequent clinical trial based on information obtained in the previous trial

	Fixed sample size design		Triangular test approach				
	One-sided type I error (Hypothetical)*	Two-sided type I error	Single triangular test			Double triangular test	
Total sample size			θ	Median**	P90**	Median	P90
Without subgroup analysis (SD = 5.3 days)	309	392	θ_A_	251	407	251	407
			θ_A_/2	265	418	272	418
			0	181	315	245	360
			−θ_A_	98	150	251	407
Pneumonia subgroup (SD = 4.6 days)	233	295	θ_B_	190	307	190	307
			θ_B_/2	200	315	205	315
			0	136	237	184	271
			−θ_B_	74	113	190	307
Bronchiolitis subgroup (SD = 5.9 days)	383	486	θ_C_	312	505	312	505
			θ_C_/2	329	518	337	518
			0	224	391	303	446
			−θ_C_	112	186	312	505

A difference of 1.5 days on a ventilator is considered clinically relevant. For the triangular test approach, sample size estimates for four different true effect sizes (θ) are given. θ_A_= 1.5/5.3; θ_B_= 1.5/4.6; θ_C_= 1.5/5.9.

SD = standard deviation

* A one-sided type I error in comparing dexamethasone with placebo in young children is considered unethical. Data are for illustration only.

** Median and P90 (90th percentile) of expected terminal sample size.

An alternative explanation for the large variance in the duration of mechanical ventilation might have been a difference in clinical routines between centres involved in this multi-centre study. No central protocol for extubation was used. If the mean duration of mechanical ventilation varied between centres irrespective of the treatment, and no correction for centre was applied, this would increase the variability of the outcome even if the treatment effect, the mean difference in duration of ventilations between the dexamethasone and placebo groups, was equal in these centres. That is why the randomization in a multi-centre trial should always be stratified by centre, and in the analysis adjustment for centre should be considered. A simulated example of the application of this principle is given in the Appendix (in Supporting Information online). Although adjustment for covariates such as centre effects is generally recommended, the sensitivity of the estimated effects to covariate adjustment has to be investigated, especially in case of dichotomous or survival outcomes (10,11).

### 2 Prevention of non-conclusive results

As we have seen, the main cause of the non-conclusive results was the unduly optimistic assumption of variance in the power calculation in the design of the trial. Apart from the possible explanations mentioned above, i.e. the existence of etiological subgroups or the omission of adjusting for centre effects, the SD estimate of 2 days based on a sub-sample of patients in an earlier study may have been inadequate due to sampling variation or due to a true difference between patients and/or interventions in the current and the former trial (12). However, it may also be due to what is sometimes called the ‘sample size samba’ (13). This term refers to the process of changing the assumptions of the power calculation in such a way that it yields a sample size that is considered desirable or feasible. When we recalculated the pooled SD for the subgroup of ventilated children in the first trial by Van Woensel et al. (9), this was shown to be 3.6 days, almost twice the assumption of 2 days used in the power calculation. Thus it seems that in this case the sample size samba has at least partly had a hand in the undue optimism of the sample size calculation of the second trial.

Obviously this could have been prevented by several measures, of which the most important in our opinion is the critical review of the design of the trial including the sample size calculation and its assumptions by clinical and statistical experts, either in the phase of review of the grant application or, when funding has been assigned, by setting up a data monitoring committee. If there is insufficient information to base the assumptions on, an internal pilot study design with sample size recalculation should be considered (14).

### 3 Could this result have been obtained more efficiently?

The results of the triangular tests using the original trial's assumptions are shown in Figure 1a–c. Assuming an inspection interval of five patients, the trial would have been stopped without rejection of the null-hypothesis after the analysis of the outcome in 55 patients, resulting in a mean difference in duration, adjusted for the sequential analysis of 2.5 days (95% CI −0.6 to 5.8 days). The result would then be indeterminate – just as in the original trial. However, it would have been generated with fewer included patients. In the bronchiolitis subgroup (Fig. 1b) the triangular test would have led to continuation of the inclusion beyond the analysis of 35 patients, probably resulting in rejection of the null-hypothesis, in favour of dexamethasone. In Figure 1c the sample path in the pneumonia subgroup can be seen to cross the middle boundaries, leading to non-rejection of the null-hypothesis, after analysis of the data from 35 patients instead of 41 in the original trial. It should be noted that these post-hoc analyses of the subgroups are merely intended for illustration to compare with the results of the original analysis and should have been corrected for multiple testing. When comparing Figures 1b and 1c the subgroups indeed appear to have a different response to dexamethasone, since the sample path almost leads to rejection of the null-hypothesis after the analysis of 35 patients in the bronchiolitis subgroup – and in effect would lead to rejection of the null-hypothesis after analysis of all 39 patients in this group, the mean difference being 4.68 [95% CI 0.62–8.68]– and to non-rejection of the null-hypothesis after analysis of 35 patients in the pneumonia subgroup.

If the empirically found SD of 5.3 days was used to design the triangular test, it would have looked like the graph shown in Figure 2. As can be seen from this graph, the sample path after the analysis of the outcome data from 80 patients would not even be near one of the boundaries, and many more patients would have to be included to obtain a definite result. The theoretical maximum number of patients to be included, indicated by the right end of the triangle would be 606, which is considerably more than the number of 392 resulting from the power calculation for the classic fixed-sample trial. However, from simulations of trial paths it can be shown that the median expected number of patients to be included before crossing one of the boundaries is 251; the 90th percentile of the expected sample size being 407 (6). The estimated sample sizes for the triangular test in the subgroups are presented in Table 1. If the true effect size deviates from the assumptions, the expected sample size in a triangular test varies accordingly, which means that the trial will be stopped earlier or later. As an illustration the medians and P90 of the sample sizes for different true effect sizes have been included in Table 1.

The triangular test is not the only possible approach to enhance efficiency in RCTs. Many different statistical approaches have been developed to enable researchers to take repeated looks at the accumulating data without inflating the pre-specified type I error. The careful evaluation of the result of such interim analyses by an independent data monitoring committee may lead to the advice to stop including patients in the trial, either because the effect of the treatment is larger than expected, e.g. (15), or because the study, if conducted until the fixed sample size is attained, will very probably not lead to rejection of the null-hypothesis, i.e. futility, e.g. (16).

### 4 How to proceed with this research?

The first step in assessing the evidence on the effect of an intervention is to assemble all the evidence that is yet available in a systematic review. If the available evidence is non-conclusive, i.e. not showing a clear beneficial or detrimental effect or a fairly narrow CI around the neutral value indicating ‘no effect’, a new trial should be designed in which the probability of obtaining definite results that support clinical decision-making is maximized. All available information about types of outcome measures and their variance, effects that are considered clinically relevant and possible subgroups should be critically evaluated and incorporated in the design of the new trial. If there is insufficient information about the variance of an outcome measure in the prospective study population, and the number of eligible patients is small, an adaptive design can be adopted, containing an internal pilot. This is an efficient way to optimize the number of patients in a trial (17,18). An internal pilot can either be used in combination with a classical approach or with a triangular test design (19). In all instances in which either an internal pilot or interim analyses are foreseen, an independent data monitoring committee should be set up before the start of the trial.

In the Cochrane Library a systematic review on the effect of glucocorticoids for acute viral bronchiolitis in infants and young children is available (20). However, in this systematic review all studies on mechanically ventilated patients were excluded. Therefore, a systematic review on the effect of glucocorticoids for acute viral bronchiolitis in ventilated infants and young children should be done. If the results of this review show that there is still insufficient evidence to rule out a detrimental, beneficial or no effect, a new trial has to be performed in the intensive care setting to generate sufficient evidence for clinical decision-making. The information on variance and effect size derived empirically in the previous trial should be used for the power calculation of this new trial. Attention should be paid in design and analysis to possible differences in clinical routines between centres as shown in the Appendix (in Supporting Information online).

The design should also assure a sufficient sample size and stratified randomization to perform a subgroup analysis of the bronchiolitis and pneumonia subgroups using an interaction test. The post-hoc subgroup analysis that was done can only be used for hypothesis-generation (21). Theoretically, for efficiency reasons one-sided hypothesis testing might be considered if the possibility of inferiority of dexamethasone is very unlikely or if clinicians are only interested in using dexamethasone if it shows a considerable improvement. This would lead to a considerable reduction of the fixed sample size as can be seen in Table 1. However, considering the result in the pneumonia subgroup, and considering the lack of trust of many clinicians in dexamethasone, in this particular case two-sided testing would be preferred.

## DISCUSSION

In this paper we present a real-life trial case to illustrate the importance of evaluating the assumptions of a power calculation. This is a very common problem, even in clinical trials reported in medical journals of ‘high’ reputation (12). The estimated number of 60 patients to be included in this trial resulting from an initial sample size calculation turned out to be a gross underestimation of the necessary sample size of 392 when using the empirically derived variance of the outcome data, i.e. days on ventilator. Based on data from a comparable earlier single-centre trial, in which SDs of duration of mechanical ventilation varied between treatment groups from 2.9 to 4.2 days, the SD of 2 days used in the power calculation for this more recent trial was found to be unduly optimistic (9).

A standardized effect size (ratio of clinically relevant difference and SD) of 0.75 is generally considered to represent a large treatment effect (22). This should have been considered beforehand. Moreover, it is not surprising that an outcome measure, which is predominantly determined by clinicians, such as duration of mechanical ventilation, varies more in a multi-centre trial than in a single centre, due to differences in local clinical routines.

Apart from attempts to explain the unexpected variance in the trial that served as a case in point, and suggesting improvement of the analysis by adjusting for centre effects, we emulated a triangular test design, using the raw data from the sample trial. This type of design would have yielded comparable results while including fewer patients.

The decision whether to use a fixed sample design or a triangular test approach to determine the number of patients that are to be included in a trial depends on the weighing of several arguments, which cannot be simply solved by a statistician in a quantitative way. It has been shown in a series of simulations that the average sample size needed to complete a trial using a sequential method in simulations was always smaller than that of the corresponding fixed design, irrespective of the effect size or power (23). However, there are several arguments of a cultural nature against the use of triangular tests. The arguments to decide between a fixed sample design and a triangular test approach are presented in Table 2. Apart from the practical and ethical issue of minimizing the sample size, there are other arguments, such as the risk of bias, the practical feasibility in a multi-centre trial, and the familiarity and acceptance of the design in the international scientific community. In spite of the general trend of these ‘qualitative arguments’ to favour the fixed sample design, the triangular test approach should be considered when useful evidence is needed for the treatment of conditions that are rare or occur in patients whose partaking in research should be minimized such as children. Because of the vulnerability of children as trial subjects, it is recommended to set up a data safety and monitoring committee for every paediatric clinical trial, even if no interim analyses or sequential methods are applied (24).

**Table 2:** Issues in the decision to choose either a fixed sample size design or a triangular test approach in a randomized clinical trial

	Fixed sample size	Triangular test
Risk of biased end result	Small if conducted according to well-known standards; if no interim analyses have been planned, analysis can be done by investigators after all trial data have been assembled	Data-analysis should be independent from trial performance and masked; important that confidentiality of results is assured until the end of the trial
Feasibility in a multi-centre trial	Logistics are known in advance; planning for a specific number of trial patients; outcome information may be assembled per centre and sent to coordinating centre later	Number of patients to be included unknown at study onset; planning may be hampered by uncertainties; block-randomization necessary to avoid discrepancies in numbers per arm; outcome information has to be sent to coordinating centre immediately when it occurs or is measured
Familiarity, acceptance by funders, editors, peers and readers	Very well-known, generally accepted as the most valid design to answer questions of effects of interventions	Less familiar design; despite unjust suspicion for increased risk of type I errors, final analysis is valid, maintaining type I error and power

When considering the necessary sample sizes as shown in Table 1, some discouragement is imaginable. To co-ordinate such a large multi-centre trial is quite a challenge. An alternative would be to conduct a so-called prospective meta-analysis, in which each centre conducts a separate randomized clinical trial, including comparable patients and using identical interventions and outcome measures (25). This way the technical possibility of pooling the data in a meta-analysis is assured, without the complicated process of a centrally led multi-centre trial.

In the present case a subgroup in a previous trial was used as a pilot for the present trial (9). It would have been more efficient for either a fixed sample design or a triangular test to use the data of the first 10 or 20 patients as an internal pilot to calculate the variance of the outcome measure without de-masking the allocation of the patients. The required sample size or the boundaries, in case of a fixed sample design or a triangular test, respectively, can then be established without doing any interim analyses (14,17). Since this was a multi-centre trial, the statistical analysis should have included investigation of a centre effect and if necessary adjustment for this.

## RECOMMENDATIONS

Issues of design and analysis of clinical trials are related to a broad range of expertise, both clinical and statistical. Therefore we think that clinical investigators should do their best to obtain critical review of their plans from the very start of the design phase until publication of the study report, and even beyond that. Almost all clinical researchers have to deal with well-known existing review institutions, which are not always appreciated because of their perceived bureaucracy and time-consuming procedures, including funding agencies and their reviewers, institutional review boards and journal editors and peer reviewers. Nevertheless, critical review and advice should also be sought from an independent data monitoring committee, both in the design phase and during the data gathering and analysis, to ensure optimal quality of the conduct of the trial and minimization of bias and random error.

## CONCLUSION

The design and analysis of the clinical trial featured in this paper has been shown to suffer from several flaws. Not only was the original power analysis unduly optimistic but also the omission of adjustment for centre effects has contributed to the lack of statistical power. These types of errors are not uncommon (26). Apart from these methodological issues, there remains the problem of how to obtain results that can serve to support clinical decision-making in case of a limited number of eligible patients.

If eligible patients are scarce, as is the case with children mechanically ventilated for RSV, it is important to make efficient use of all available information. If the international medical scientific community recognizes the importance of obtaining evidence for the treatment of rare diseases, often the case in paediatrics, the possible methodological alternatives to large randomized clinical trials have to be explored and taken seriously (27). This recognition will also have to be earned by researchers in this field conducting and reporting trials in a transparent and methodologically sound way, and making maximum use of the expertise of critical clinicians and statisticians in data monitoring committees.

## SUPPORTING INFORMATION

**Appendix** Dealing with heterogeneous health outcome subgroups within a multicentre trial.

**Appendix Table S1** Overall and centre specific mean ventilation durations and standard deviations (SD) for placebo and dexamethasone treated patients as observed in the simulated data.

**Appendix Table S2** Estimated simulated overall means, standard error of the mean (SEM) and 95% confidence interval per treatment and for the difference based on a model without or with adjustment for centre from the simulated data.
