# Supplementary material for: Pitfalls in the design and analysis of paediatric clinical trials: a case of a ‘failed’ multi-centre study, and potential solutions
Source: Acta Paediatr. 2009 Feb;98(2):385–91. doi: 10.1111/j.1651-2227.2008.01048.x (PMC2659390; doi:10.1111/j.1651-2227.2008.01048.x)
Supplement: Supplementary file 1 [file apa0098-0385-SD1.doc]

**Appendix (Supplementary Material online)**

**Dealing with heterogeneous health outcome subgroups within a multicentre trial**

To demonstrate the effect of heterogeneous subgroups within a population, a simulation was carried out. For the simulated trial, we assumed that data were collected in five centres using a randomized block design with 80 patients in total, i.e. 8 patients per treatment arm per centre. For each patient in this hypothetical trial, a ventilation time (the primary outcome) was randomly drawn from a multivariate normal distribution. This multivariate normal distribution was created using a model that assumed a beneficial effect of dexamethasone treatment on the duration of the mechanical ventilation of 1.5 days. Furthermore, we assumed that, irrespective of the treatment received, the average mechanical ventilation duration differed between centres. The simulated average ventilation durations were 6, 7, 7, 17 and 17 days for centre 1 to 5, respectively. The overall standard deviation was set at 2 days.

The observed overall and centre specific mean ventilation durations in the simulated data for placebo and dexamethasone treated patients are shown in Table 1 and the results of the statistical analysis of these data using either a model without (t-test) or with (two-way ANOVA) correction for centre are shown in Table 2.

On average, treatment with dexamethasone reduced the ventilation duration with 1.4 days in our simulated data. However, due to a large standard error, as a result of the heterogeneous subgroups, this difference was not statistically significant. The resulting 95% CI indicates that dexamethasone might reduce the ventilation duration with 3.7 days, but also increase it with almost a day.

When the centre differences are taken into account, the average gain is estimated with more precision resulting in a 95% CI that excludes zero days: dexamethasone significantly reduces the ventilation duration with, on average, 1.4 days. However, depending on the centre where the patient is treated, the ventilation duration on dexamethasone treatment can still vary between 4 and 16 days.

**(Supplementary Material online)**

Appendix Table 1: Overall and centre specific mean ventilation durations and standard deviations (SD) for placebo and dexamethasone treated patients as observed in the simulated data.

| Overall | Observed mean (days) | SD |
| --- | --- | --- |
| Placebo (n = 40) | 11.5 | 5.3 |
| Dexamethasone (n = 40) | 10.1 | 5.0 |
|  |  |  |
| Per centre (n = 8 per treatment per centre) | Observed mean (days) | SD |
| Centre 1 Placebo | 5.8 | 1.7 |
| Dexamethasone | 4.2 | 2.2 |
| Centre 2 Placebo | 8.0 | 1.9 |
| Dexamethasone | 6.7 | 2.1 |
| Centre 3 Placebo | 9.0 | 1.9 |
| Dexamethasone | 8.6 | 2.4 |
| Centre 4 Placebo | 18.1 | 2.1 |
| Dexamethasone | 14.6 | 1.4 |
| Centre 5 Placebo | 16.5 | 1.7 |
| Dexamethasone | 16.3 | 1.3 |

Appendix Table 2: Estimated simulated overall means, standard error of the mean (SEM) and 95% confidence interval per treatment and for the difference based on a model without or with adjustment for centre from the simulated data.

| Without adjustment for centre | | | | |
| --- | --- | --- | --- | --- |
| Treatment | Mean (days) | SEM | 95% confidence interval | |
| Placebo | 11.5 | 0.8 | 9.8 | 13.1 |
| Dexamethasone | 10.1 | 0.8 | 8.5 | 11.7 |
| Gain due to treatment | 1.4 | 1.2 | -0.9 | 3.7 |
| p-value for difference: 0.228 | | | | |
|  | | | | |
| With adjustment for centre | | | | |
| Treatment | Mean (days) | SEM | 95% confidence interval | |
| placebo | 11.5 | 0.3 | 10.9 | 12.1 |
| Dexamethasone | 10.1 | 0.3 | 9.5 | 10.7 |
| Gain due to treatment | 1.4 | 0.4 | 0.5 | 2.3 |
| p-value for difference: 0.002 | | | | |
